# HDAC1 and HDAC2 independently regulate common and specific intrinsic responses in murine enteroids

**DOI:** 10.1038/s41598-019-41842-6

**Published:** 2019-03-29

**Authors:** Alexis Gonneaud, Naomie Turgeon, Christine Jones, Cassandra Couture, Dominique Lévesque, François-Michel Boisvert, François Boudreau, Claude Asselin

**Affiliations:** Département d’anatomie et biologie cellulaire, Faculté de médecine et des sciences de la santé, Pavillon de recherche appliquée sur le cancer, Université de Sherbrooke, Sherbrooke, Québec J1E 4K8 Canada

## Abstract

Both HDAC1 and HDAC2 are class I deacetylases acting as erasers of lysine-acetyl marks on histones and non-histone proteins. Several histone deacetylase inhibitors, either endogenous to the cell, such as the ketogenic β-hydroxybutyrate metabolite, or exogenous, such as butyrate, a microbial-derived metabolite, regulate HDAC activity. Different combinations of intestinal epithelial cell (IEC)-specific *Hdac1* and/or *Hdac2* deletion differentially alter mucosal homeostasis in mice. Thus, HDAC1 and HDAC2 could act as sensors and transmitters of environmental signals to the mucosa. In this study, enteroid culture models deleted for *Hdac1* or *Hdac2* were established to determine IEC-specific function as assessed by global transcriptomic and proteomic approaches. Results show that *Hdac1* or *Hdac2* deficiency altered differentiation of Paneth and goblet secretory cells, which sustain physical and chemical protection barriers, and increased intermediate secretory cell precursor numbers. Furthermore, IEC *Hdac1*- and *Hdac2*-dependent common and specific biological processes were identified, including oxidation-reduction, inflammatory responses, and lipid-related metabolic processes, as well as canonical pathways and upstream regulators related to environment-dependent signaling through steroid receptor pathways, among others. These findings uncover unrecognized regulatory similarities and differences between *Hdac1* and *Hdac2* in IEC, and demonstrate how HDAC1 and HDAC2 may complement each other to regulate the intrinsic IEC phenotype.

## Introduction

The small intestinal epithelium is composed of a single row of epithelial cells divided in proliferative crypt and differentiated villus compartments^1^. Crypt-located reserve intestinal stem cells sustain epithelial renewal by dividing in columnar stem cells generating transit-amplifying cells. These cells further segregate in absorptive enterocytes and secretory progenitor cells, precursors of Paneth, goblet and enteroendocrine cells^2^. Each differentiated cell lineage contributes to small intestinal functions, notably by establishing physical and chemical barriers between the host and the luminal diet and microbial content, and by providing a sensing and transmitting interface between the lumen and the mucosal immune system^3^. Indeed, in addition to absorptive and digestive functions, enterocytes, the most abundant intestinal epithelial cells (IEC), achieve selective barrier permeability through tight junction interactions between intestinal epithelial cells^4–6^. Enterocytes also participate in the chemical barrier by expressing transmembrane mucins as well as cytokines and antimicrobial proteins, in response to the microbial environment^7^. Goblet cells produce the mucus layer preventing bacterial adhesion to the epithelium as well as various antimicrobial proteins, and deliver luminal antigens to dendritic cells^6,8^. Crypt-located Paneth cells support the stem cell niche and produce different constitutive or inducible antimicrobial proteins to insure epithelial protection^9,10^. Many signaling pathways, including the Wnt and Notch pathways, regulate intestinal stem cell maintenance, renewal and differentiation^11,12^. Intestinal homeostasis is secured by interdependent communication signals between the intestinal mucosal system along with IEC, the luminal environment with diet-derived and microbial products, as well as the microbiota. However, alterations in the intestinal environment or the immune system, in conjunction with genetic susceptibilities, may lead to intestinal defects, including inappropriate inflammatory responses^3^.

Environmental changes are transmitted to the cell through epigenetic modifications of histones^13^. Acetylation is one epigenetic signal implicated as environmental sensor. Acetyltransferase readers add an acetyl group to histones on lysines, leading to alterations in DNA-histone interactions or to the production of acetyl marks recognized by bromodomain-containing regulators^14^. Histone acetylation efficiency is regulated in part by variations in mitochondrial and nucleo-cytosolic acetyl-CoA levels as a result of the cellular metabolic state^15,16^. Lysine acetylation is also controlled by histone deacetylase (HDAC) erasers that remove acetyl groups from histones and non-histone proteins. Endogenous HDAC activity is inhibited by metabolites including β-hydroxybutyrate^17,18^, L-carnitine^19^ and sphingosine-1-phosphate^20^, as well as diet- and bacterially-derived metabolites, such as butyrate^21–24^. Among HDACs, HDAC1 and HDAC2 are zinc-dependent class I deacetylases associated with Sin3A, CoREST and NuRD protein complexes regulating transcription, DNA replication and DNA repair, among others^25–27^. *Hdac1* deletion in mice leads to embryonic lethality^28^ while *Hdac2* deficiency results in perinatal lethality stemming from heart defects^29^. In contrast to no apparent defects or subtle phenotypic alterations in many *Hdac1* or *Hdac2* tissue-specific deletion models, dual *Hdac1* and *Hdac2* deficiency triggers extensive differentiation and proliferation alterations in most tissues^25^. Of note, gene-dosage experiments in mice have indicated different sensitivities to *Hdac1* or *Hdac2* expression levels. For example, murine brain is altered in mice with one allele of *Hdac1* without *Hdac2* in neural cells, as opposed to neural cells with one allele of *Hdac2* without *Hdac1*^30^. Similarly, differentiation is altered in mice with one allele of *Hdac2* without *Hdac1* in epidermal cells, as opposed to epidermal cells with one allele of *Hdac1* without *Hdac2*^31^.

In the intestine, IEC-specific *Hdac1* and *Hdac2* villin-Cre-induced deletion results in increased proliferation, goblet and Paneth cell loss, polarity disruption, activation of Notch, Stat3 and mTOR pathways, as well as increased susceptibility to DSS-induced colitis^32,33^. While *Hdac2* IEC-specific deletion does not alter intestinal homeostasis, *Hdac2* deficiency protects against DSS-induced colitis^33^. In addition, short-term deletion of both *Hdac1* and *Hdac2* in IEC with the Ah-Cre model leads to proliferation arrest^34,35^, accompanied by DNA damage responses^35^. Finally, in contrast to mice with one *Hdac1* allele without *Hdac2*, villin-Cre mice with one allele of *Hdac2* without *Hdac1* display homeostatic defects similar to double villin-Cre *Hdac1* and *Hdac2* knockout phenotypic alterations^35^.

These results suggest that HDAC1 and HDAC2 display redundant and specific functions in intestinal epithelial cells that could alter IEC interactions with the environment, leading to modifications in intestinal homeostasis. In this study, to determine the intrinsic IEC-specific role of HDAC1 and HDAC2, we have established enteroid culture models deleted for *Hdac1* or *Hdac2* and assessed the IEC phenotype by transcriptomic and proteomic approaches. We show that *Hdac1* or *Hdac2* deficiency alters differentiation of secretory cells that sustain physical as well as chemical protection barriers, and increases the number of intermediate secretory cell precursors. We identify IEC *Hdac1*- and *Hdac2*-dependent common and specific biological processes, including oxidation-reduction and lipid-related metabolic processes, as well as canonical pathways and upstream regulators related to environment-dependent signaling through steroid receptor pathways, among others. These findings uncover unrecognized regulatory similarities and differences between *Hdac1* and *Hdac2* in IEC, and demonstrate how HDAC1 and HDAC2 may complement each other to regulate the intrinsic IEC phenotype.

## Results

**Figure 1:**
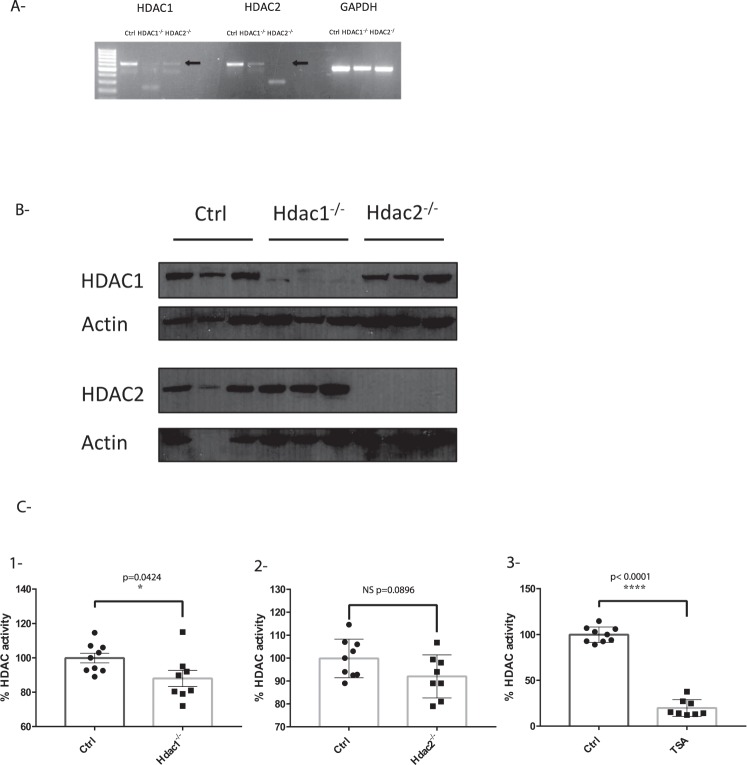
*Hdac1* or *Hdac2* depletion reduces enteroid histone deacetylase activity. (**A**) Total RNA was isolated from 5-day cultured control, *Hdac1*- and *Hdac2*-deficient enteroids. *Hdac1* and *Hdac2* mRNA levels were determined by semi-quantitative RT-PCR, with *Gapdh* as a loading control. (**B**) Total protein extracts from control, *Hdac1*- and *Hdac2*-deficient enteroids were separated on SDS-PAGE gels for Western blot analysis, and selected proteins were revealed with specific antibodies against HDAC1 and HDAC2, and against β-ACTIN as a loading control. Cropped images for HDAC1 and β-ACTIN are from immunoblotting experiments on the same membrane. Cropped images for HDAC2 and β-ACTIN are from immunoblotting experiments on another membrane. Full-length blots are presented in Supplementary Fig. S8 (n = 3). (**C**) 7.5 µg of nuclear proteins from control, *Hdac1*- and *Hdac2*-deficient enteroids were used to measure deacetylase activity with a colorimetric HDAC assay kit. Treatment with Trichostatin A, a pan-inhibitor of HDAC activity, was used as a control (n = 3; 2 or 3 wells for each). Results represent the mean ± SD (*p ≤ 0.05; ****p ≤ 0.0001).

To determine the intrinsic effect of HDAC1 and HDAC2 on IEC, we generated enteroid cultures from isolated jejunal crypts of villin-Cre control, *Hdac1* and *Hdac2* knockout mice^32^. RT-PCR (Fig. 1A) and Western blot analysis (Fig. 1B), as well as immunofluorescence (Supplementary Fig. S1) confirmed *Hdac1* or *Hdac2* deletion at both the RNA and protein levels in the respective enteroid cultures. As opposed to other cell models with *Hdac1* or *Hdac2* deletions^25^, enteroid-specific *Hdac1* or *Hdac2* deletion did not lead to significantly increased protein levels of the remaining HDAC (Fig. 1B). As assessed by a colorimetric assay, we observed significantly reduced nuclear deacetylase activity in *Hdac1*-depleted enteroids (12% decrease, p = 0.0424), but no significant differences in HDAC activity in *Hdac2*-depleted enteroids (p = 0.0896), as opposed to a 80.2% HDAC activity reduction upon TSA treatment (Fig. 1C).

**Figure 2:**
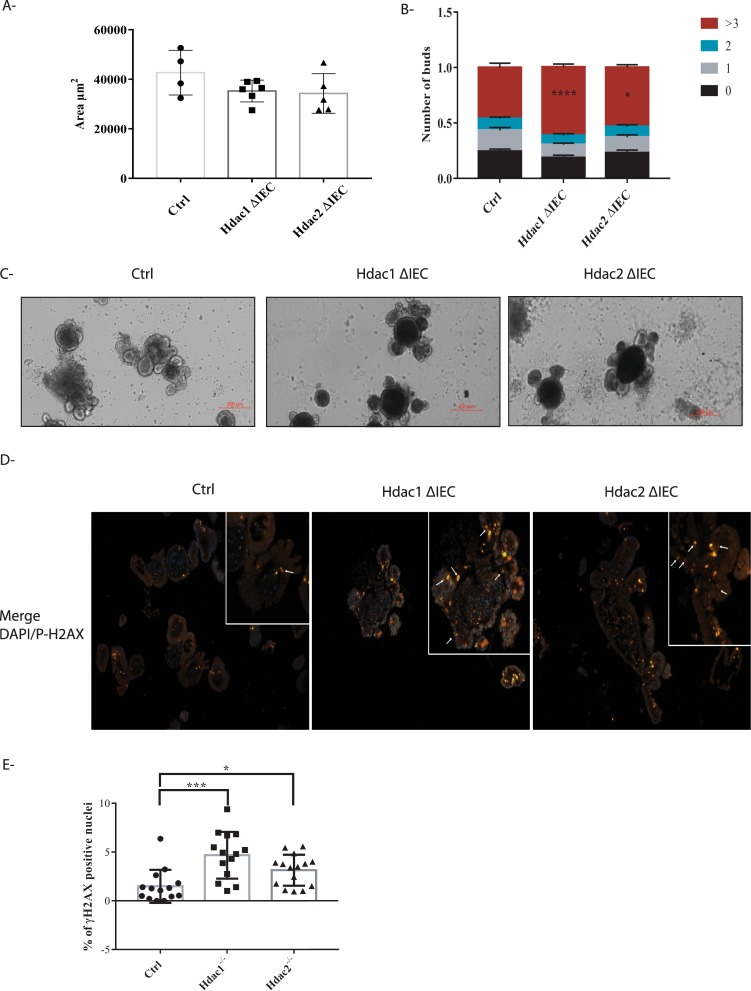
*Hdac1* or *Hdac2* depletion differently alters enteroid homeostasis. (**A**) Surface measurement of 5-day cultured control, *Hdac1*- and *Hdac2*-deficient enteroids (n = 3; 3–6 wells, and 50–70 enteroids for each). (**B**) Structural assessment of 5-day cultured control, *Hdac1*- and *Hdac2*-deficient enteroids by bud number scoring (n = 3; 3–6 wells for each): no bud (sphere), one bud, two buds, three or more buds. Results represent the mean ± SEM (*p ≤ 0.05; ****p ≤ 0.001 for more than 3 buds). (**C**) Representative micrographs of 5-day cultured control, *Hdac1*- and *Hdac2*-deficient enteroids. The scale bar indicates the relative size (bar = 200 μm). (**D**) Representative images of control, *Hdac1*- and *Hdac2*-deficient enteroids labeled with an antibody against phosphorylated γ-H2AX. Nuclei are stained with DAPI. γ-H2AX labeled cells are indicated by arrows. Magnification: 10X  . For inserts, Magnification: 20X. (**E**) Number of γ-H2AX labeled nuclei in *Hdac1*- or *Hdac2*-depleted enteroids relative to control enteroids (n = 2). 15 independent fields (total surface: 7.8 mm^2^) (n = 2). Results represent the mean ± SD (*p ≤ 0.05; ***p ≤ 0.005).

While there were no statistical differences in enteroid size, as assessed by surface area measurement (Fig. 2A; p > 0.1, one-way ANOVA), loss of *Hdac1* or *Hdac2* resulted in differences in enteroid structure, as determined by enhanced crypt budding per enteroid (Fig. 2B,C). Proliferation was not significantly altered, as assessed by BrdU pulse labeling (Supplementary Fig. S2). As HDAC1 and HDAC2 regulate DNA repair induced by double-strand breaks (DSB)^36^, we verified the expression of the DSB repair marker, γ-H2AX. A significant increase in the number of γ-H2AX stained nuclei in both *Hdac1*- or *Hdac2*-deficient enteroids was observed by immunofluorescence with a phospho-γ-H2AX antibody (Fig. 2D,E; Supplementary Fig. S3).

**Figure 3:**
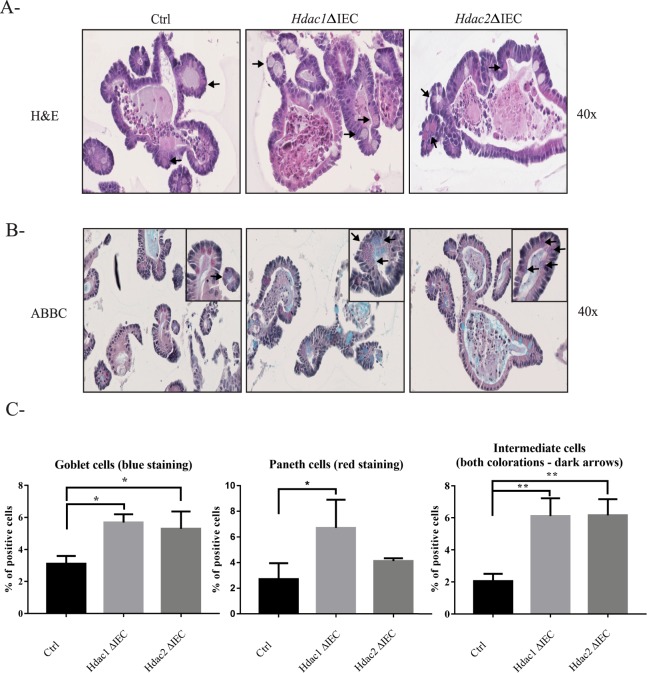
*Hdac1* or *Hdac2* depletion modifies enteroid differentiation. Control, *Hdac1*- and *Hdac2*-deficient enteroid sections were stained with Hematoxylin and Eosin (H&E) (**A**) or with a combination of Alcian blue and Best’s Carmine, respectively for goblet cells and Paneth cells (**B**). Magnification: 40X. For inserts, Magnification: 60X. Arrow: intermediate cell. (**C**) Number of goblet cells, Paneth cells and intermediate cells in *Hdac1*- or *Hdac2*-depleted enteroids relative to control enteroids (n = 3, 25–35 enteroids per experiment). Results represent the mean ± SEM (*p ≤ 0.05; **p ≤ 0.01).

**Figure 4:**
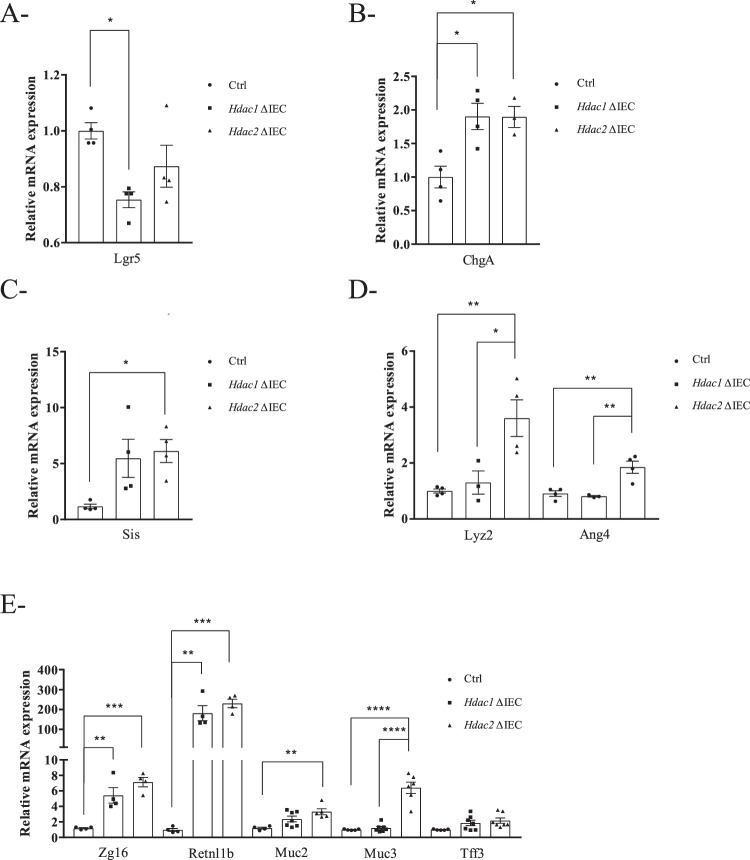
*Hdac1* or *Hdac2* depletion alters enteroid differentiated gene expression levels. Total RNA was isolated from 5-day cultured control, *Hdac1*- and *Hdac2*-deficient enteroids. Expression levels of stem cell marker *Lgr5* (**A**), enteroendocrine cell marker *ChgA* (**B**), enterocyte marker *Si* (**C**), Paneth cell markers *Lys2* and *Ang4* (**D**), and goblet cell markers *Zg16*, *Retnlb*, *Muc2*, *Muc3*, *Tff3* (**E**) were determined by qPCR, with *Pbgd* as a control (n = 4–7). Results represent the mean ± SEM (*p ≤ 0.05; **p ≤ 0.01; ***p ≤ 0.005; ****p ≤ 0.001).

To determine the intrinsic effect of *Hdac1* or *Hdac2* deletion on enteroid differentiation, H&E staining demonstrated an increase in the numbers of goblet cells associated with granules, in both enteroid mutants (Fig. 3A, see arrows and insert). Simultaneous staining with Alcian Blue to label goblet cells, and with Best’s Carmine to label Paneth cells, showed a significant increase in the number of goblet cells in both mutant enteroids, and in the number of Paneth cells in *Hdac1*-deleted enteroids (Fig. 3B,C). A significant augmentation in the number of intermediate cells displaying both goblet and Paneth cell labels was also observed (Fig. 3C, arrows and insert; Supplementary Fig. S4). Intermediate cells are considered precursors of both Paneth and goblet cell lineages in the small intestine^37,38^. The expression of differentiation markers was measured by qPCR. Different patterns of gene expression were observed in mutant organoids. While mRNA expression of stem cell marker *Lgr5* was decreased in *Hdac1*-deficient enteroids (Fig. 4A), the expression of the enteroendocrine cell marker *ChgA*, as well as goblet cell markers *Zg16* and *Retnlb* was increased in both *Hdac1*- or *Hdac2*-depleted enteroids (Fig. 4B,E). Augmented expression of enterocyte gene *Sis*, Paneth cell markers *Lyz2* and *Ang4*, and goblet cell markers *Muc2* and *Muc3* was observed in *Hdac2*-deficient enteroids, in contrast to *Hdac1*-deleted enteroids. These data suggest that epithelial differentiation is altered after *Hdac1* or *Hdac2* deletion in enteroids, and that HDAC1 and HDAC2 are not equivalent in their regulatory function.

**Figure 5:**
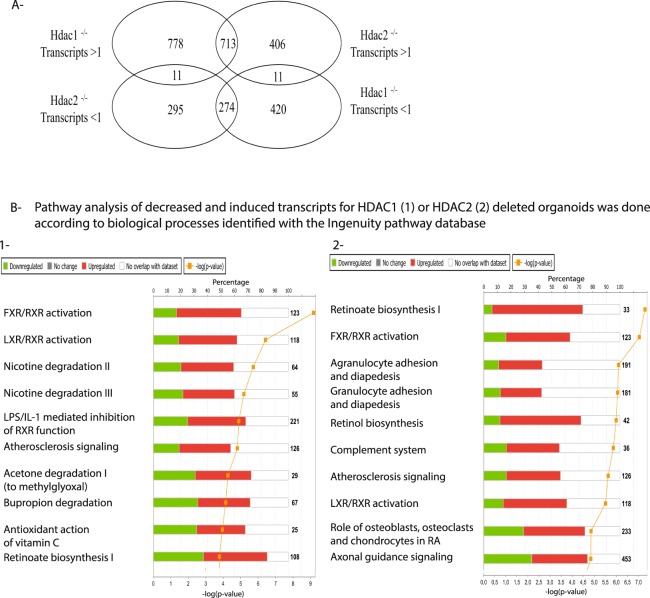
Transcript changes in *Hdac1*- or *Hdac2*-deficient enteroids, and top canonical pathways. (**A**) Venn diagram illustrating the specificity and the overlap of increased or decreased gene expression patterns between *Hdac1*-deficient and *Hdac2*-deficient enteroids (P-value < 0.05; log_2_ > 1 and log_2_ < 1), as assessed by RNA-Seq analysis. Top canonical pathways for gene expression identified by IPA analysis are shown for *Hdac1*-deficient enteroids (**B-1**) and *Hdac2*-deleted enteroids (**B-2**).

Having demonstrated specific and similar alterations between *Hdac1*- and *Hdac2*-deficient enteroids, we then determined global patterns of RNA expression by RNA-Seq, selecting log_2_ > 1 and log_2_ < 1 *Hdac1* or *Hdac2* specific genes with DESeq adjusted p-value ≤ 0.05. Based on this selection, RNA-Seq analysis revealed 1491 and 1119 genes respectively increased in *Hdac1*- and *Hdac2*-deficient enteroids, as well as 694 and 569 genes separately decreased (Fig. 5A, Supplementary Tables S5, S6). Increased or decreased expression of respectively 713 and 274 genes overlapped with both *Hdac1*- and *Hdac2*-deficient enteroids. FXR/RXR activation was a common top canonical pathway while lipopolysaccharide represented a common top upstream regulator, as determined by IPA analysis (Fig. 5B, Supplementary Tables S1, S2). Bioinformatics analysis revealed other specific top canonical pathways (LXR/RXR activation, Nicotine degradation II and III, LPS/IL-1 mediated inhibition of RXR function) in *Hdac1*-depleted enteroids. Additional specific predicted top upstream regulators were either inhibited and related to *Ppara* (ACOX1, ciprofibrate), or activated and related to *Rara* signaling (tretinoin) (Supplementary Table S1). Likewise, other top canonical pathways for *Hdac2*-depleted enteroids included retinoate biosynthesis I and retinol biosynthesis, with TNF as an activated top upstream regulator (Fig. 5B, Supplementary Table S2).

Biological processes identified by Gene Ontology analysis with DAVID identified common up-regulated processes, including inflammatory response, response to lipopolysaccharide, extracellular matrix organization, oxido-reduction process, lipid transport and retinol metabolic process (P-value between 3.80E-06 and 1.70E-03) (Supplementary Fig. S5). Common down-regulated processes included multicellular organism development and angiogenesis (P-value between 2.40E-08 and 1.20E-03). Specific increased biological processes included cholesterol homeostasis, superoxide and lipoprotein metabolic process for *Hdac1*-depleted enteroids (P-value between 8.10E-05 and 1.10E-03), while negative regulation of cell proliferation and negative regulation of cell migration were decreased (P-value of 9.8E-05 and 2.10E-03). Likewise, specific increased biological processes included response and defense response to virus for *Hdac2*-depleted enteroids (P-value between 1.90E-05 and 1.70E-04), while response to interferon-gamma, positive regulation of gene expression and of DNA-templated transcription were decreased (P-value between 6,10E-04 and 3.10E-03).

**Figure 6:**
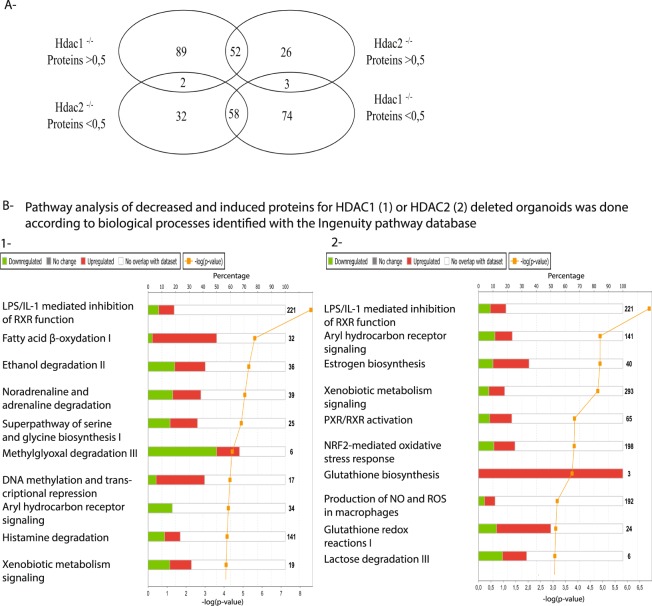
Protein changes in *Hdac1*- or *Hdac2*-deficient enteroids, and top canonical pathways. (**A**) Venn diagram illustrating the specificity and the overlap of increased or decreased protein expression patterns between *Hdac1*-deficient and *Hdac2*-deficient enteroids (P-value < 0.05; log_2_ > 1 and log_2_ < 1), as assessed by SILAC proteomics analysis. Top canonical pathways for protein expression identified by IPA analysis are shown for *Hdac1*-deficient enteroids (**B-1**) and *Hdac2*-deleted enteroids (**B-2**).

Changes in global protein expression between wild type and mutant enteroid cells were then quantified by quantitative mass spectrometry after SILAC labeling, selecting >1.5-fold and <1.5-fold specific proteins identified by at least two peptides, with an FDR 5%. Proteomic analysis revealed 141 and 78 proteins respectively increased in *Hdac1*- and *Hdac2*-deficient enteroids, as well as 132 and 90 proteins separately decreased in *Hdac1*- and *Hdac2*-deleted enteroids (Fig. 6A, Supplementary Table S7). Increased or decreased expression of respectively 52 and 58 proteins overlapped in both mutated enteroids.

LPS/IL-1 mediated inhibition of RXR function was a common top canonical pathway while CFTR represented a common top upstream regulator, as determined by IPA analysis (Fig. 6B). Further specific top canonical pathways were revealed in *Hdac1*- (Fatty acid oxidation I, Ethanol degradation) and *Hdac2*-depleted enteroids (Aryl hydrocarbon receptor signaling, Estrogen biosynthesis, Xenobiotic metabolism signaling, PXR/RXR activation) (Fig. 6B), in addition to predicted top upstream regulators associated with *Hdac1*- (PPARA, CFTR, PPARG, Essra, IL10RA) and *Hdac2*-deficient enteroids (CFTR, NFE2L2, IL10RA) (Fig. 6B, Supplementary Tables S3, S4). While not predicted as activated, top upstream regulators identified in *Hdac2*-deficient enteroid proteomes included steroid receptors such as NR1I3 and PXR ligand-PXR-retinoic acid-RXR.

Biological processes identified by Gene Ontology analysis with DAVID included common up-regulated *Hdac1*- and *Hdac2*-dependent processes, such as oxidation-reduction and lipid metabolic process (P-value between 2.40E-15 and 3.1E-03) (Supplementary Fig. S6). Common down-regulated processes included glutathione metabolic process, metabolic process, cell-cell adhesion and oxido-reduction process (P-value between 8.20E-05 and 3.10E-03). Specific increased biological processes included fatty acid, retinoid, carbohydrate, cholesterol, lipoprotein and steroid metabolic processes, as well as lipid and cholesterol transport for *Hdac1*-depleted enteroids (P-value between 2.90E-07 and 4.30E-04), while ATP-dependent chromatin remodeling, RNA splicing, mRNA processing and retinoic acid metabolic processes were decreased (P-value between 7.10E-04 and 4.90-E03). Likewise, specific increased biological processes included response to drug, nutrient, and hormone, among others, for *Hdac2*-depleted enteroids (P-value between 1.40-05 and 1.10E-03), while responses to different stimuli were decreased (P-value between 9.60E-04 and 3.70E-02). Oxido-reduction process was the GO term shared by the transcriptome and the proteome of both *Hdac1*- and *Hdac2*-depleted enteroids. Thus, HDAC1 and HDAC2 may both regulate, to different extent, metabolic and oxido-reduction processes, as well as cell responses to endogenous and exogenous environmental metabolites, in part through steroid receptor signaling.

**Figure 7:**
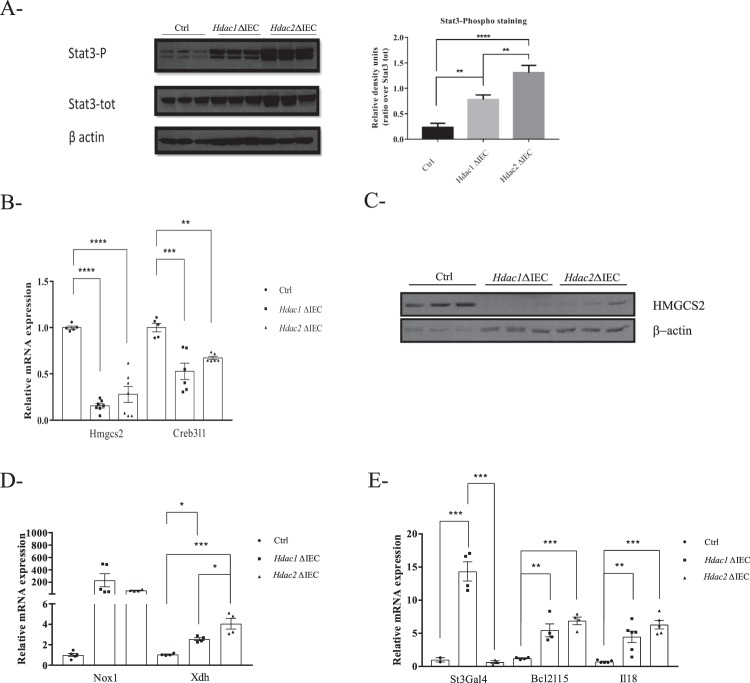
*Hdac1* or *Hdac2* depletion alters the expression of specific metabolic and inflammatory related genes. Total protein extracts from control, *Hdac1*- or *Hdac2*-deficient enteroids were separated on SDS-PAGE gels for Western blot analysis, and selected proteins were revealed with specific antibodies against phosphorylated and total STAT3 (**A**), against HMGCS2 (**C**), and against β-ACTIN as a loading control (**A**,**C**) (n = 3). Densitometric analysis of Phospho-STAT3 compared to STAT3 is indicated (**p ≤ 0.01; ****p ≤ 0.001). Cropped images for Phospho-STAT3, STAT3 and β-ACTIN are from immunoblotting experiments on the same membrane. Cropped images for HMGCS2 and β-ACTIN are from immunoblotting experiments on another membrane. Samples for (**A**,**C**) derive from the same experiments. Full-length blots are presented in Supplementary Fig. S8. (**A**). Total RNAs were isolated from 5-day cultured control, *Hdac1*- or *Hdac2*-deficient enteroids. Expression levels of *Hmgcs2* and *Creb3l1* (**B**), *Nox1* and *Xdh* (**D**), *St3gal4*, *Bcl2l15 and Il18* (**E**) were determined by qPCR, with *Pbgd* as a control (n = 4–7). Results represent the mean ± SEM (*p ≤ 0.05; **p ≤ 0.01; ***p ≤ 0.005; ****p ≤ 0.001).

Proteome and transcriptome analysis revealed common activation of STAT signaling in *Hdac1*- and *Hdac2-*deficient enteroids. Indeed, STAT1 was the top transcriptional regulator identified by RNA-Seq analysis while IL10RA was one of the top upstream regulators identified by proteome analysis. Since an increased Stat3 phosphorylation can be observed in dual *Hdac1* and *Hdac2* IEC-specific deleted mice^32^, Stat3 phosphorylation levels were verified in mutated enteroids. The data show an increase in Stat3 phosphorylated forms in *Hdac1*- as well as *Hdac2*-deleted enteroids (Fig. 7A).

Based on common and specific biological processes revealed by RNA-Seq and proteomic analysis, we selected a subset of genes associated with metabolism, stress and environmental responses, for further verification of gene expression patterns by qPCR. Expression of *Hmgcs2*, a mitochondrial enzyme involved in the ketogenesis pathway and regulated in part by PPARA^39^, was decreased at both mRNA and protein levels (Fig. 7B,C). Expression of *Creb3l1*, an unfolded protein response regulator^40^, was decreased in *Hdac1*- or *Hdac2*-deficient organoids, as assessed by qPCR (Fig. 7B). Increased expression of *Nox1*, a NADPH oxidase^41^, *Xdh*, involved in purine oxidative metabolism^42^, *Il18*, a proinflammatory cytokine induced by inflammasome signaling^43^, and *Bcl2l15*, an apoptosis regulator expressed at high levels in the intestine^44^, was observed (Fig. 7D,E). Other genes encoding *Nfkbiz*, a regulator of NF-κB activity^45^, *Nos2*, a nitric oxide synthase^46^, and *Apoa4*, an apolipoprotein^47^, displayed increased expression in both *Hdac1*- and *Hdac2*-deficient enteroids while expression of *Dusp4*, a MAP kinase phosphatase^48^, was decreased (Supplementary Fig. S7). Expression of *St3gal4*, a glycosyltransferase^49^, was increased specifically in *Hdac1*-deficient enteroids (Fig. 7E). These results indicate common and specific patterns of gene regulation by *Hdac1* or *Hdac2* deficiency in enteroids, which affect cell-intrinsic metabolic as well as cell-extrinsic environmental responses.

## Discussion

In order to determine the IEC intrinsic role of histone deacetylase HDAC1 and HDAC2, we have performed a global phenotypic, transcriptomic and proteomic analysis of *Hdac1*- and *Hdac2*-deficient enteroids. Our results show that *Hdac1* or *Hdac2* deletion in enteroids leads to increased production of secretory Paneth and goblet cells, suggesting a contribution of HDAC1 and HDAC2 in the regulation of Notch signaling. Indeed, active Notch signaling prevents secretory cell differentiation^12,50^. We have also observed increases in the number of intermediate cells, considered as precursors of both Paneth and goblet cell lineages in the small intestine^37,51^. Notch signaling is also involved in the production of intermediate cells. Indeed, intermediate as well as secretory cell numbers are increased in the small intestine of mice treated with the Notch pathway inhibitor DBZ^38^. Intestinal epithelial-specific deletion of the Adam10 sheddase gene in mice leads to increased numbers of Paneth/goblet cell intermediates as well as enteroendocrine cells through Notch signaling regulation^52^.

*In vivo*, we have previously observed that IEC-specific *Hdac1*- or *Hdac2*-deficient mice do not display intestinal architectural defects. However, in contrast to *Hdac1*^+/−^; *Hdac2*^*−/−*^ IEC-deficient mice, IEC-deleted *Hdac1*^*−/−*^; *Hdac2*^*−/−*^ or *Hdac1*^*−/−*^; *Hdac2*^+/−^ are characterized by IEC loss of polarity and barrier function, resulting in a chronic inflammation, concomitant with increased crypt proliferation, decreased secretory cell numbers and Notch pathway activation^32,35^. These data suggest that different HDAC1 and HDAC2 levels may alter the balance between stem and progenitor cell proliferation, and differentiation, in part by regulating Notch pathway activity. *In vivo*, some additional contributions from the intestinal environment may differentially regulate intestinal homeostasis. Further studies to compare *Hdac1*^+/−^; *Hdac2*^*−/−*^ or *Hdac1*^*−/−*^; *Hdac2*^+/−^ enteroids to single *Hdac1-* or *Hdac2-*deleted organoids, and to assess their responses to mucosal environment factors, including cytokines, could be performed. Nevertheless, our data indicate that HDAC1 and HDAC2 may regulate intestinal epithelial cell fate, in part through Notch pathway regulation, as well as the relative production and maturation of differentiated cells from progenitors.

An increase in the number of phosphorylated γ-H2AX foci is observed after *Hdac1* or *Hdac2* deletion. HDAC1 and HDAC2 play important roles in DNA repair and the DNA damage response to double-strand breaks^36,53^, major inducers of γ-H2AX phosphorylation^54^. HDAC1 and HDAC2 are also recruited to replication forks^55^ to regulate DNA replication^56^. It is thus possible that *Hdac1* or *Hdac2* deletion renders enteroids more sensitive to DNA-strand breaks or replication stresses, leading to increased phosphorylated γ-H2AX foci. Recent data suggest that changes in chromatin structure *per se* may also result in γ-H2AX phosphorylation. For example, hypotonic treatment of different cell lines leads to increased γ-H2AX foci formation, in the relative absence of DNA strand-breaks^57^. Thus, *Hdac1* or *Hdac2* deletion could induce the formation of γ-H2AX foci by affecting chromatin structure, in part through histone acetylation.

Our data indicate that HDAC1 or HDAC2 homodimers, while sharing some targets, are not functionally equivalent to HDAC1/HDAC2 heterodimers in HDAC1/HDAC2 co-repressor complexes^25^. *Hdac1* or *Hdac2* deletion could lead to global effects on histone acetylation patterns, resulting in transcriptional changes. It has been proposed that HDAC associated with actively transcribed gene promoters could be important in resetting active promoters through deacetylation, for further rounds of transcription^58,59^. While increased histone acetylation correlates with active gene expression, it was shown that HDAC inhibition with HDAC pharmacological inhibitors, which results in increased histone acetylation in gene bodies and intragenic domains, may hinder transcriptional elongation and eRNA-associated enhancer transcription, suggestive of a positive role in transcriptional elongation^60^. Whether specific *Hdac1* or *Hdac2* deletion in enteroids could affect the expression of a subset of genes at the level of elongation or enhancer/promoter usage remains to be determined.

Our transcriptomic and proteomic analysis has uncovered various HDAC1- or HDAC2-dependent pathways acting as internal or external signaling sensors or transmitters. For example, HDAC1 and HDAC2 may play a role in the production of retinoic acid (RA), as suggested by increased Retinol metabolic processes in mutant enteroids. IEC-derived RA is considered as an important mucosal signal to mediate immune responses^61^. Of note, IEC-derived RA production is increased by the bacterial-derived short-chain fatty acid butyrate, a HDAC inhibitor, as well as by HDAC pharmacological inhibitors^62,63^.

IPA and GO term analysis of transcriptome and proteome data have revealed shared pathways regulated by *Hdac1* or *Hdac2*. *Hdac1* and *Hdac2* deletion alters inflammatory signaling pathways. For example, STAT1 and RELA are in the top transcription factor upstream regulator, as well as IL10RA, suggesting the implication of HDAC1 and HDAC2 in respectively IFN signaling^64^, in cytokine-mediated NF-κB signaling^65^ and in STAT3 activation by cytokines^66^. HDAC may directly regulate the acetylation levels of these transcription factors, and their activity. For example, HDAC1 and HDAC2 negatively regulate NF-κB^65^. Class I HDAC, including HDAC1 and HDAC2, regulate STAT1 as well as STAT3 acetylation and transcriptional activity^64^. We have shown an increase in STAT3 phosphorylation in *Hdac1*- and *Hdac2*-deleted enteroids. This induced phosphorylation could be indirect, through autocrine production of cytokines, or direct as STAT3 acetylation may promote STAT3 phosphorylation, leading to increased transcriptional activity^64,67^. STAT3 is an important downstream effector of many cytokine-induced pathways which regulate IEC barrier function and mucosal wound healing^68^. For example, IL-22-dependent induction of STAT3 is essential for IEC barrier integrity and epithelial regeneration after injury, both *in vivo* and in organoid cultures^69^. Thus, HDAC1 and HDAC2 regulation of STAT3 activity may play an important role to insure intestinal homeostasis.

IPA and GO term analysis of transcriptome and proteome data have also revealed specific pathways regulated by *Hdac1* or *Hdac2*. *Hdac1* deletion leads to PPARα pathway inhibition, as a top upstream regulator, and to alterations in many metabolic pathways related to lipid and fatty acid metabolic processes. PPARα activation by fatty acids ligands, among others, regulates fatty acid metabolism, through peroxisomal and mitochondrial β-oxidation, inflammation and ketogenesis^39,70^. For example, expression of PPARα-regulated mitochondrial *Hmgcs2*, the rate-limiting enzyme involved in the production of ketone bodies^39,71^, including β-hydroxybutyrate, a known endogenous HDAC inhibitor^17,18^, is decreased in mutant enteroids. Thus, *Hdac1* or *Hdac2* enteroid deficiency may alter directly or indirectly the production of endogenous inhibitors, which could maintain HDAC activity.

*Hdac2*-deleted enteroids display AhR and PXR/CAR xenobiotic signaling as top canonical pathway and top upstream regulator, respectively. AhR is a transcription factor regulated by many endogenous and exogenous diet- and microbiota-derived ligands. AhR play important roles in the intestinal mucosal immune response and the regulation of the intestinal barrier function, as well as in antioxidant and xenobiotic responses^72,73^. AhR signaling is increased after HDAC inhibitor treatment of cultured murine and human IEC^74^. In addition to their role as xenobiotic sensors, recent data have uncovered roles for CAR and PXR in energy homeostasis^75^, as well as a role for PXR in intestinal epithelial repair responses^76^.

In addition to metabolic processes, oxido-reduction is one of the top processes affected in *Hdac1*- and *Hdac2*-deleted enteroids. This suggests that lack of HDAC1 or HDAC2 may affect enteroid metabolism. Acetyl-CoA levels vary according to the cellular metabolic state, with high levels associated with cell growth and survival, and global histone acetylation^77^. Acetyl-CoA is generated in various cellular compartments, including the nucleus, the cytoplasm and mitochondria. In the nucleus, the acetyl group linked to lysines on histones may supply acetate, in order to restore nuclear acetyl-CoA levels. Changes in HDAC activity could affect acetate availability, and thus influence enteroid homeostasis through metabolic pathway alterations, as proposed before^78^. Indeed, we have previously found that *Hdac1* depletion in the intestinal epithelial cell line IEC-6 alters metabolic processes^79^. Whether metabolic process modifications observed in *Hdac1*- and *Hdac2*-deleted enteroids are indeed a compensatory response to maintain proper acetyl-CoA levels remains to be determined.

The extensive transcriptomic and proteomic changes observed upon *Hdac1* or *Hdac2* deletion in enteroids may be direct, through lysine acetylated mark alterations in regulatory regions, or indirect. This could occur either through acetylation-dependent alterations of the function of non-histone proteins, including transcription factors such as STAT3^80^, or by metabolite changes that could result from decreased acetate availability. To identify more specific targets, chromatin immunoprecipitation experiments for specific acetylated marks, HDAC1 and HDAC2 could be performed with, for example, inducible *villin*-Cre^ER^ *Hdac1*- or *Hdac2*-deleted enteroids. Mass spectrometry experiments could be carried out in order to assess the changes in the acetylome of *Hdac1*- or *Hdac2*-depleted enteroids.

We have identified here, homeostatic pathways dependent on HDAC1 and HDAC2 activity in enteroids. Our data suggest that HDAC1 and HDAC2 regulate differentiation, barrier function and stress responses in enteroids. Furthermore, HDAC1 and HDAC2 in part regulate common pathways related to oxydo-reduction and inflammatory processes in enteroids. Specific pathways include lipid metabolism for HDAC1 and response to environmental signals for HDAC2. Nuclear HDAC activity may well be important to control cellular acetyl-CoA levels necessary to insure enteroid homeostasis. Thus, IEC-intrinsic modifications of HDAC1 and/or HDAC2 activity may mediate the intestinal mucosal responses observed in IEC-specific *Hdac1*- and/or *Hdac2*-deficient mice^32,33,35^. As specific HDAC inhibitors are developed for use in cancer and in various immune and neurological diseases^81,82^, analysis of their impact on global and specific effects of HDAC1 and HDAC2 should be considered.

## Methods

### Enteroid culture

*Hdac1* and *Hdac2* floxed mice^29^ were crossed with villin-Cre transgenic mice^83^ in a C57BL/6 J X 129SV X CD1 background. All experiments were performed in accordance with relevant guidelines and regulations, and were approved by the Institutional Animal Research Review Committee of the Université de Sherbrooke (protocol 360-14B)^33,35^. Genomic DNA was isolated with the Spin Doctor genomic DNA kit (Gerard Biotech) to determine the genotypes^33,35^. Jejunal crypts from one wild-type, *Hdac1* or *Hdac2* mutant mouse were isolated by EDTA fractionation and cultured according to previous publications^84,85^, and as we have done before^86^. Matrigel (Growth Factor Reduced, BD Corning) embedded enteroids were grown in ENR medium containing 70% Advanced DMEM/F-12 Flex medium (Thermo Fisher Scientific), 1.25 mM N-acetylcysteine (Sigma), 50 ng/mL EGF (Life Technologies), B27 supplement 1X (Life Technologies), N2 supplement 1X (Life Technologies), 10% Advanced DMEM/F-12 Noggin conditioned medium and 20% Advanced DMEM/F-12 R-Spondin 1 conditioned medium. Penicillin (100 U/mL) and streptomycin (100 µg/mL) were also added to the medium. Enteroids were passaged every 5 days by mechanical disruption, and embedded 1:3 in 20 µL Matrigel (about 10 enteroids per µL).

### Enteroid characterization

Wild-type, *Hdac1*- and *Hdac2*-deficient enteroid surface areas were measured after 5 days in culture (n = 3; 3 to 6 wells for each). Perimeters to measure enteroid areas were determined from horizontal cross-sections with the ZEISS ZEN Microscope Software. Budding efficiencies were measured under light microscopy by scoring for the number of buds per enteroid after 5 days: no budding (0), one bud (1), two buds (2) and three or more buds (3+). Imaging was performed with Cell Discoverer 7 microscope (magnification: 10X) (Zeiss, Toronto, ON, Canada).

### HDAC activity measurement

Matrigel was dissolved in ice-cold Cell Recovery solution (Corning), and nuclear protein extracts were prepared from recovered wild-type, *Hdac1*- and *Hdac2*-deficient enteroids grown for 5 days, by using Abcam nuclear extraction kit (ab113474). 7.5 µg of nuclear extracts were used to measure nuclear HDAC activity with the colorimetric Epigenase HDAC activity/inhibition direct assay kit (Epigentek), on a Versamax ELISA microplate reader at 450 nm (Molecular Devices), according to the manufacturer’s protocol (n = 3; 2–3 wells for each). As a control, nuclear extracts were incubated with the HDAC pan-inhibitor Trichostatin A (1 μM). Results are expressed as the mean ± SD. Statistical significance was determined by Student’s *t*-test.

### Histological analysis and immunofluorescence

Five-day wild-type, *Hdac1*- or *Hdac2*-deleted enteroid cultures were used. Enteroids recovered from Matrigel were separated by mechanical disruption, centrifuged for 3 min at 7000 rpm, and suspended in 4% paraformaldehyde for 1 h at 4^o^C. Fixed enteroids were centrifuged at 7000 rpm for 3 min, suspended in 70% ethanol for 1 h at 4^o^C and centrifuged at 7000 rpm for 3 min. Enteroids were mixed with Histogel (Thermo Fisher Scientific) before paraffin inclusion^32,33^. Sections were stained with hematoxylin and eosin for histological analysis, with Alcian blue for goblet cell mucins and with Best’s Carmine for Paneth cells. Goblet, Paneth and intermediate cell numbers in *Hdac1*- or *Hdac2*-depleted enteroids relative to control enteroids (n = 3, 25–35 enteroids per experiment) were counted in a blinded fashion, by two independent investigators. For immunofluorescence experiments, paraffin-embedded enteroid sections were rehydrated with graded ethanol series and boiled for 6 min in 10 mM citric acid. Treated sections were then blocked in PBS supplemented with 0.1% BSA and 0.2% Triton for 45 min^86^. Proliferation was assessed by fluorescein-conjugated mouse anti-bromodeoxyuridine (BrdU) (1:50, BMC 9318, Roche Diagnostics) staining of sections obtained from enteroid cultures incubated for 90 min with 10 µM BrdU. BrdU positive nuclei were counted with Cell Profiler 3.15^87^. Other primary antibodies include rabbit anti-HDAC1 (ab7028) (1:500, Abcam), rabbit anti-HDAC2 (ab7029) (1:500, Abcam), rabbit anti-phosphohistone γH2A.X (sc-101696) (1:500, Santa Cruz Biotechnology). γH2A.X positive stained nuclei were counted with Cell Profiler 3.15 (surface area for each experiment: 7.8 mm^2^, n = 2). Secondary donkey F(ab’)2 Anti-Rabbit IgG H&L (Alexa Fluor 568) preadsorbed antibodies (Abcam) were incubated at room temperature for 2 h (n = 3). Additional information about antibodies is included in Supplementary Table S8.

### Protein isolation and Western blotting

Five-day wild-type, *Hdac1*- or *Hdac2*-deleted enteroid cultures were used, unless otherwise stated. Enteroids were recovered and directly lysed in 1 X Laemmli buffer 1 (62.5 mM Tris-HCl pH 6.8, 2% SDS, 10% glycerol) supplemented with protease and phosphatase inhibitors. Whole enteroid protein content was measured with the Pierce BCA Protein Assay Kit (Thermo Fisher Scientific). Fifteen µg of total protein extracts were added on 4–12% SDS-polyacrylamide gels, and transferred on PVDF membranes (Roche Molecular Biochemicals). Western blotting was performed as described previously^79^. Membranes were incubated 1 h at room temperature or overnight at 4 °C with the following primary antibodies: rabbit anti-HDAC1, rabbit anti-HDAC2 (Abcam); rabbit anti-HMGCS2, rabbit anti-phosphoSTAT3, rabbit anti-STAT3 (Cell Signaling); mouse anti-ACTIN (EMD Millipore). Secondary antibodies included goat anti-mouse and goat anti-rabbit (Invitrogen). Immune complexes were revealed with Amersham ECL^TM^ Western blotting detection reagents (GE Healthcare) (n = 3). Additional information about antibodies is included in Supplementary Table S8.

### RNA isolation, qPCR and semi-quantitative RT-PCR

Total RNAs from wild-type, *Hdac1*- or *Hdac2*-deleted enteroids were purified with the RNeasy mini kit (Qiagen), and quantified with a NanoDrop ND-1000 spectrophotometer (Thermo Fisher Scientific). cDNAs were synthetized from 1 µg of RNA with oligodT and Superscript II reverse transcriptase (Life Technologies). For semi-quantitative RT-PCR, cDNA amplification for *Hdac1*, *Hdac2* and *Gapdh* with the Taq PCR Master Mix Kit (Qiagen, Mississauga, ON, Canada) was performed by a first 94 °C cycle for 5 min, followed by 29 cycles of 1 min at 94 °C, 45 sec starting at 62 °C and decreasing in increments of 0.3 °C every cycle, 1 min at 72 °C, and a final cycle of 1 min at 94 °C and 10 min at 72 °C (Supplementary Table S9 for PCR primer sequences). For qPCR analysis, 10 ng of cDNAs were amplified with the Brilliant III Ultra-fast SYBR Green qPCR Master Mix (Agilent Technologies) and specific gene upstream and downstream oligonucleotides (Supplementary Table S9), with a 10 min cycle at 95 °C, followed by 40 cycles of 10 sec at 95 °C, 10 sec at 60 °C and 20 sec at 72 °C. qPCR was performed with a Corbett RotorGene (Qiagen/Corbett Research). Relative RNA amounts were determined by using porphobilinogen deaminase (*Pbgd*) amplification (n = 4–7).

### RNA-Seq analysis

Total RNAs from wild-type, *Hdac1*- or *Hdac2*-deleted enteroids were purified with the RNeasy mini kit (Qiagen) (n = 4), and quantified. RNA integrity was evaluated with a 2100 Bioanalyzer (Agilent Technologies). Only samples with RNA Integrity Number (RIN) > 6.5 were selected. cDNA library preparation and transcriptome analysis with the Illumina HiSeq. 4000 PE100 sequencing system (Illumina) were performed at the McGill University and Génome Québec Innovation Center. Sequence alignment was performed with the Star 2.4.0.1 software package based on genome reference Mus_musculus:GRCm38. Differentially expressed genes were identified with DESeq adjusted p-value ≤ 0.05^88^. Only transcripts increased or decreased more than 2-fold between wild-type, *Hdac1*- or *Hdac2*-deleted enteroids were selected for further bioinformatics analysis. RNA-Seq data have been deposited in the Gene Expression Omnibus database (GSE124146).

### Proteome analysis

Wild-type, *Hdac1*- or *Hdac2*-deleted enteroids were grown for 6 passages in SILAC ENR medium supplemented with arginine and lysine, either the normal light isotopes of carbon, hydrogen and nitrogen (^12^C^14^N) (L), the medium L-arginine-^13^C_6_^14^N_4_ and L-lysine-^2^H_4_ isotopes (M) or the heavy L-arginine- ^13^C_6_-^15^N_4_ and L-lysine-^13^C_6_-^15^N_2_ isotopes (H), as done before^86^. SILAC-labeled enteroids were recovered after six days in culture. Enteroid pellets were suspended in a 10 mM HEPES pH 7.0–7.6, 8 M urea solution, and proteins were processed for HPLC-MS/MS analysis (n = 2)^86^. Briefly, proteins were reduced in 3.24 mM dithiothreitol (DTT) before being alkylated in 13.5 mM iodoacetamide. After BCA protein quantitation, pools of 15 µg of light-, medium- or heavy-isotope labeled samples were digested by trypsin. Trypsin digested peptides were separated with an Ultimate U3000 nanoflow LC-system (Dionex Corporation). The HPLC system was coupled to an OrbiTrap Q Exactive, via an EasySpray source. Spray voltage was set to 1.5 kV, with a 40 °C column temperature. Full scan MS survey spectra (m**/**z 350–1800) in profile mode were acquired in the Orbitrap with a resolution of 70,000 after accumulation of 1,000,000 ions. The ten most intense peptide ions were fragmented by collision-induced dissociation (normalized collision energy 35% with a resolution of 17,500) after the accumulation of 50,000 ions. Maximal filling times were 250 ms for the full scans and 60 ms for the MS/MS scans. Data were acquired using the XCalibur software.

SILAC-labeled proteins were identified and quantified with MaxQuant version 1.5.2.8 software^89^ with the Uniprot mouse protein database containing 89,422 proteins, to which 175 commonly observed contaminants and all the reversed sequences had been added. To achieve reliable identification, proteins with the number of forward hits in the database at least 100-fold higher than the number of reverse database hits, were accepted, thus resulting in less than 5% false discovery rate (FDR). Quantification was performed with a minimum of 2 peptides for each protein, and only in the presence of 3 ratio counts. The mass spectrometry proteomics data have been deposited to the ProteomeXchange Consortium via the PRIDE partner repository with the dataset identifier PXD011869 (http://proteomecentral.proteomexchange.org).

### Bioinformatics analysis

For both transcriptome and proteome, classification of genes according to Gene ontology was performed with the Database for Annotation, Visualization and Integrated Discovery software (DAVID 2.0)^90^ (p < 0.05), and with the Ingenuity Pathway Analysis software (IPA, Qiagen). These classifications identified altered biological processes and variations between samples, in order to predict pathway activation (URA, z-score)^91^.

### Statistical analysis

Data were expressed as means ± SEM, or SD for HDAC activity measurement. Groups were compared by Student’s t-test (unpaired), one-way ANOVA with tukey multiple comparison test.

## Supplementary information

Supplementary Table legends S1–S9

Supplementary Figures S1–S8

Supplementary Table S1

Supplementary Table S2

Supplementary Table S3

Supplementary Table S4

Supplementary Table S5

Supplementary Table S6

Supplementary Table S7

Supplementary Table S8

Supplementary Table S9
