# Supplementary material for: HDAC1 and HDAC2 independently regulate common and specific intrinsic responses in murine enteroids
Source: Sci Rep. 2019 Mar 29;9:5363. doi: 10.1038/s41598-019-41842-6 (PMC6441098; doi:10.1038/s41598-019-41842-6)
Supplement: Supplementary file 1 — Supplementary Table legends S1–S9 [file 41598_2019_41842_MOESM1_ESM.pdf]

HDAC1 and HDAC2 independently regulate common and specific intrinsic responses in murine enteroids. Alexis Gonneaud, Naomie Turgeon, Christine Jones, Cassandra Couture, Dominique Lévesque, François-Michel Boisvert, François Boudreau and Claude Asselin* Département d'anatomie et biologie cellulaire, Faculté de médecine et des sciences de la santé, Pavillon de recherche appliquée sur le cancer, Université de Sherbrooke, Sherbrooke, Québec, Canada J1E 4K8

SUPPLEMENTARY TABLE LEGENDS

Supplementary Table S1. List of *Hdac1*-depleted enteroid upstream regulators identified by IPA analysis from RNA-Seq experiments.

Supplementary Table S2. List of *Hdac2*-depleted enteroid upstream regulators identified by IPA analysis from RNA-Seq experiments.

Supplementary Table S3. List of *Hdac1*-depleted enteroid upstream regulators identified by IPA analysis from proteomics experiments.

Supplementary Table S4. List of *Hdac2*-depleted enteroid upstream regulators identified by IPA analysis from proteomics experiments.

Supplementary Table S5. List of *Hdac1*-depleted enteroid mRNA increased and decreased two-fold (p-value < 0.05), $log_2>1$ and $log_2<1$ (DESeq adjusted pvalue).

Supplementary Table S6. List of *Hdac2*-depleted enteroid mRNA increased and decreased two-fold (p-value < 0.05), $log_2>1$ and $log_2<1$ (DESeq adjusted pvalue).

Supplementary Table S7. List *Hdac1*- and *Hdac2*-depleted enteroid protein increased and decreased, in log_2, 2 peptides and more.

Supplementary Table S8. List of antibodies used for Western Blot and immunofluorescence.

Supplementary Table S9. List of oligonucleotide sequences used for qPCR and semi-quantitative RT-PCR.
