# Supplementary material for: HDAC1 and HDAC2 independently regulate common and specific intrinsic responses in murine enteroids
Source: Sci Rep. 2019 Mar 29;9:5363. doi: 10.1038/s41598-019-41842-6 (PMC6441098; doi:10.1038/s41598-019-41842-6)

HDAC1 and HDAC2 independently regulate common and specific intrinsic responses in murine enteroids. Alexis Gonneaud, Naomie Turgeon, Christine Jones, Cassandra Couture, Dominique Lévesque, François-Michel Boisvert, François Boudreau and Claude Asselin* Département d'anatomie et biologie cellulaire, Faculté de médecine et des sciences de la santé, Pavillon de recherche appliquée sur le cancer, Université de Sherbrooke, Sherbrooke, Québec, Canada J1E 4K8

SUPPLEMENTARY FIGURE LEGENDS S1-S8

Supplementary Figure S1. Control, *Hdac1*- and *Hdac2*-deficient enteroid sections were stained with DAPI and specific antibodies against HDAC1 or HDAC2. Magnification: 20 X.

Supplementary Figure S2. Quantitation (Cell Profiler 3.15) of control, *Hdac1*- and *Hdac2*-deficient enteroids labeled with BrdU (**A**) and representative images (**B**). 11 independent fields (total surface: 5.8 mm^2) (n=2). Magnification: 10 X.

Supplementary Figure S3. Control, *Hdac1*- and *Hdac2*-deficient enteroid sections were stained with DAPI and labeled with an antibody against phosphorylated γ-H2AX (additional pictures). Magnification: 10 X, but for inserts, Magnification: 20 X.

Supplementary Figure S4. Control, *Hdac1*- and *Hdac2*-deficient enteroid sections were stained with a combination of Alcian blue and Best's Carmine, respectively for goblet cells and Paneth cells (additional pictures). Magnification: 40 X, but for inserts, Magnification: 60 X. Arrow: intermediate cell.

Supplementary Figure S5. RNA-Seq Gene ontology term enrichment of biological processes for decreased and induced mRNA (fold change >2 and <2) in *Hdac1*- (**A**) and *Hdac2*-deficient enteroids (**B**). Gene ontology analysis was done using the EASE Score from DAVID 2.0 (p-value ≤ 0.05).

Supplementary Figure S6. Proteomic SILAC Gene ontology term enrichment of biological processes for decreased and induced proteins (fold change >50% and <50%) in *Hdac1*- (**A**) and *Hdac2*-deficient enteroids (**B**). Gene ontology analysis was done using the EASE Score from DAVID 2.0 (p-value ≤ 0.05).

Supplementary Figure S7. ***Hdac1* or *Hdac2* depletion alters the expression of specific metabolic and inflammatory related genes.** Total RNA was isolated from 5-day cultured control, *Hdac1*- or *Hdac2*-deficient enteroids. Expression levels of *Nfkbiz*, *Dusp4*, *Nos2* and *Apoa4* were determined by qPCR, with *Pbgd* as a control (n=4-7). Results represent the mean ± SEM (*p≤0.05; ** p≤0.01; *** p≤0.005).

Supplementary Figure S8. Full-length blots for Figure 1B, Figure 7A and Figure 7C.

Supplementary Figure S1

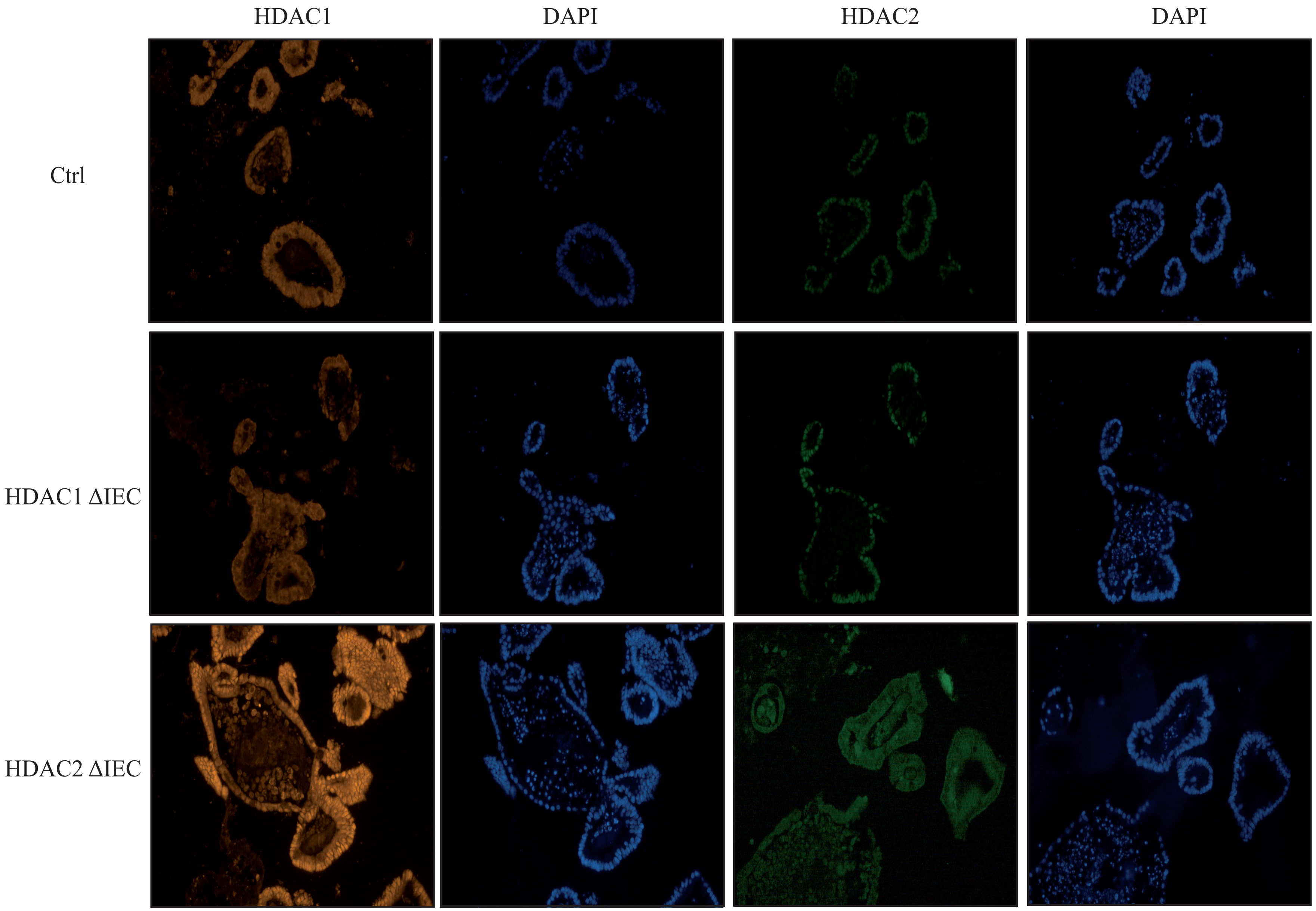

Supplementary Figure S2

A-

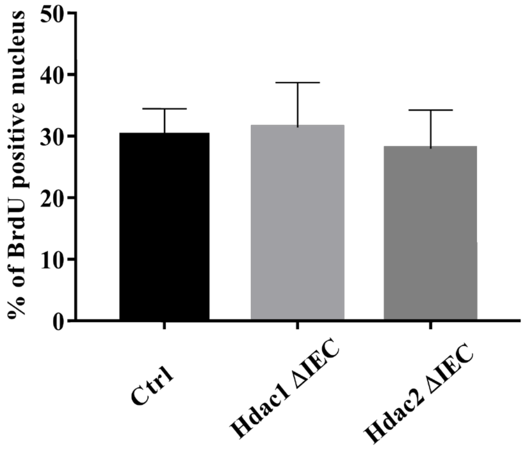

B-

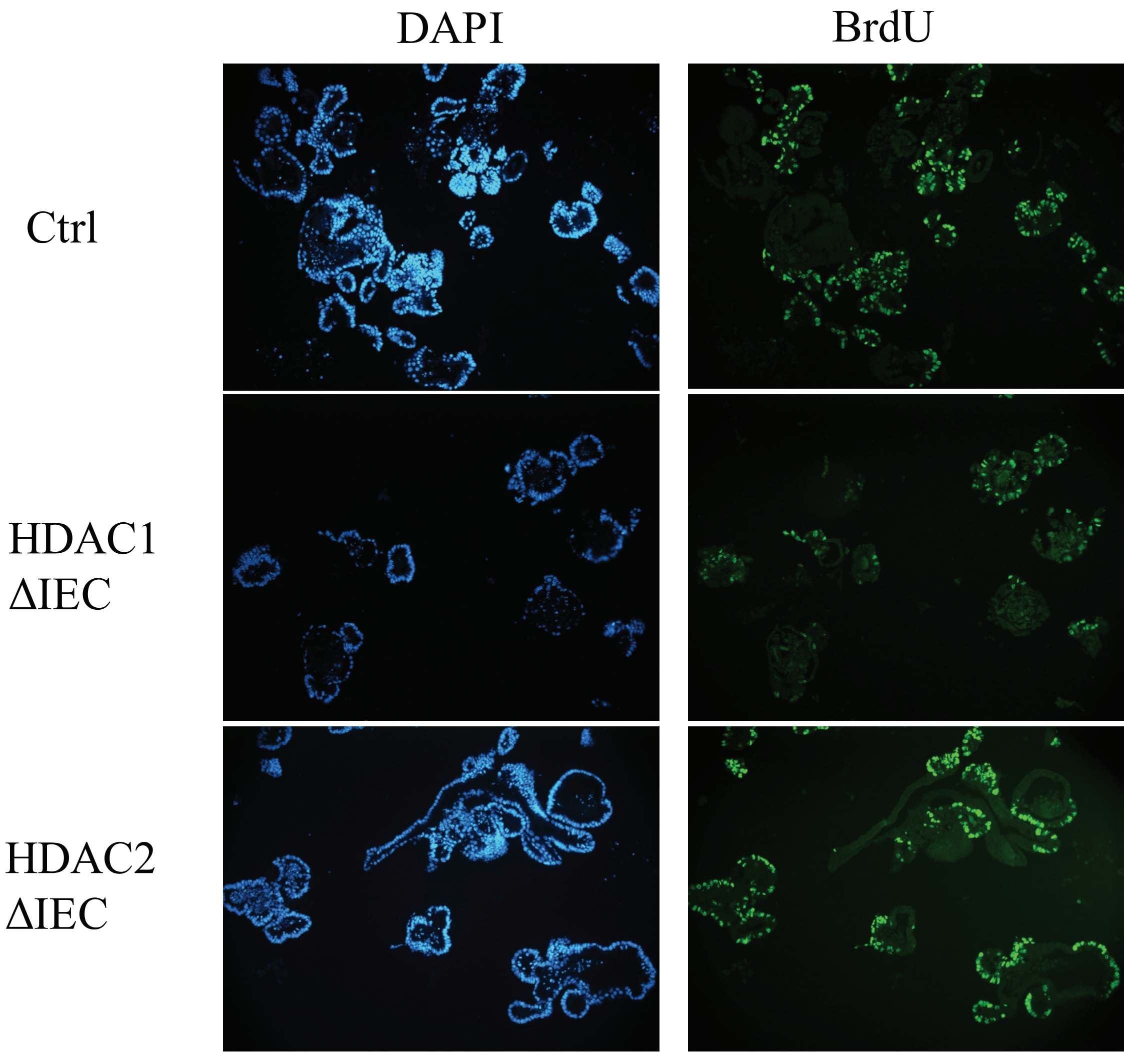

Supplementary Figure S3

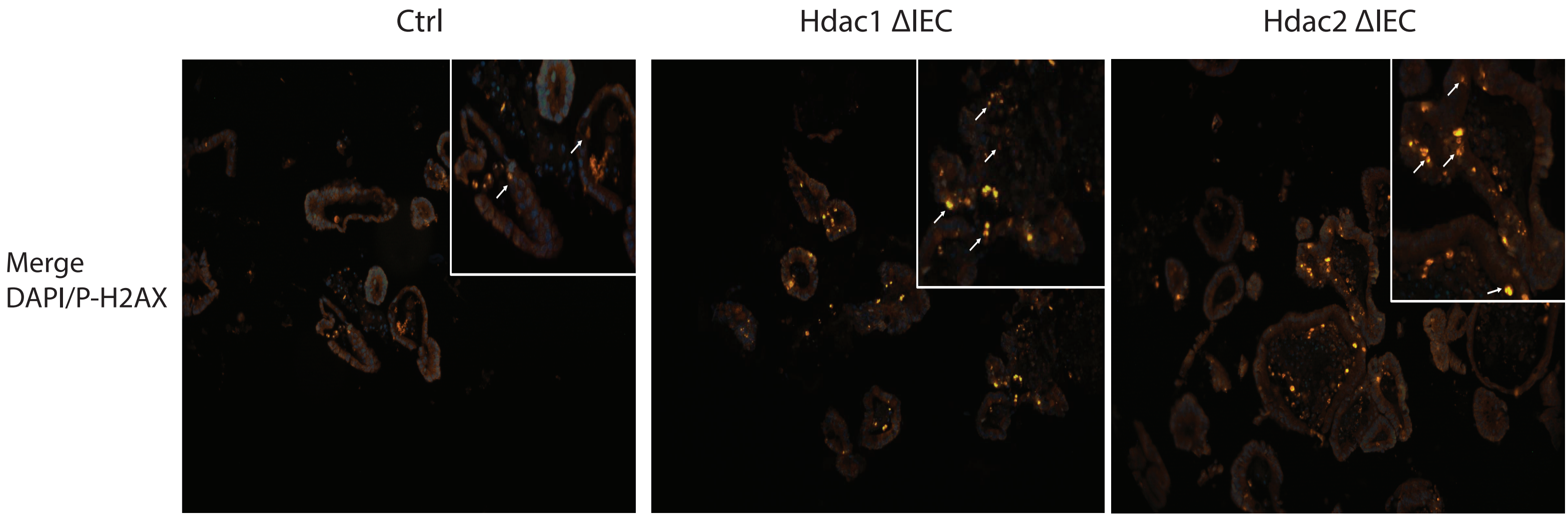

Supplementary Figure S4

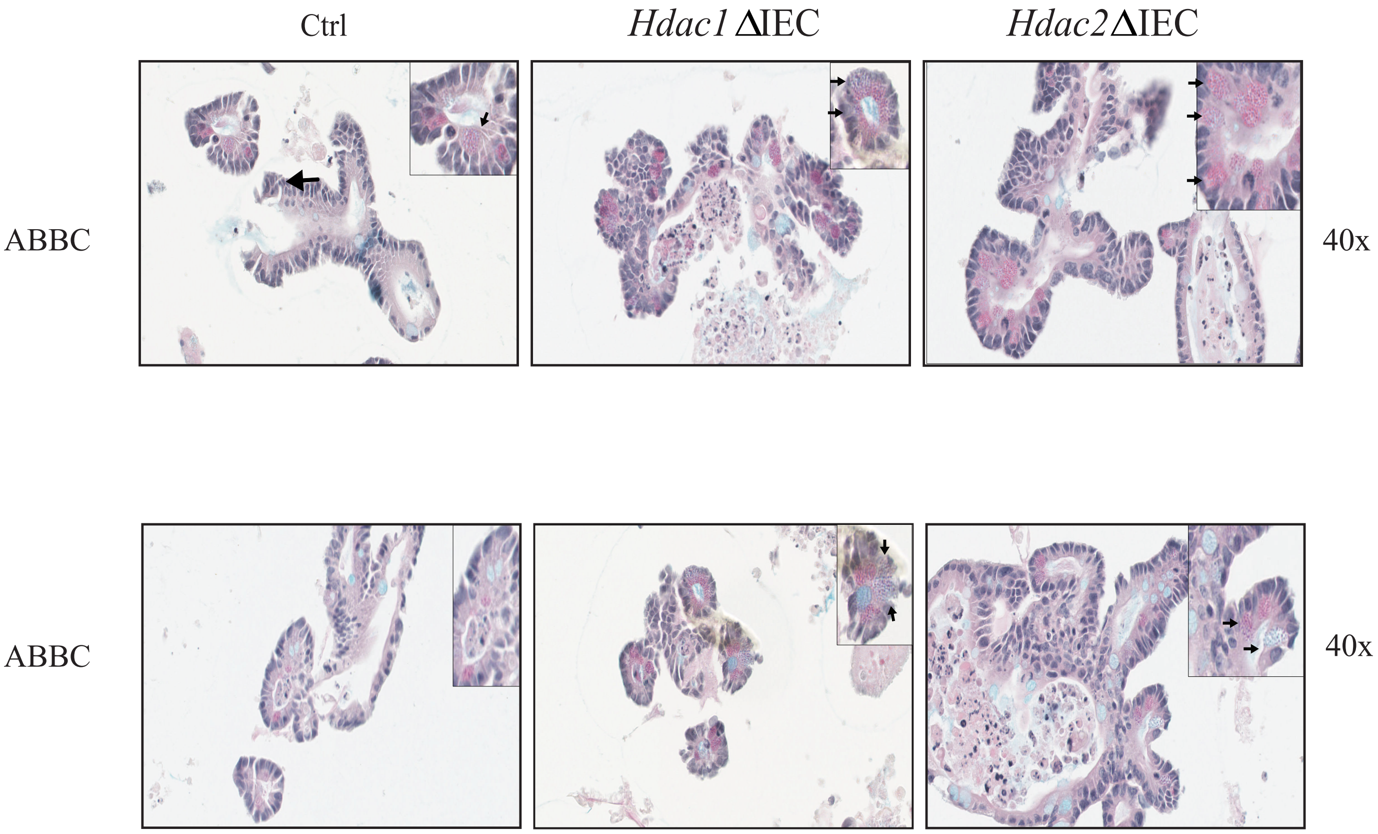

Supplementary Figure S5

A-

GOTERM_BP_DIRECT of *Hdac1* deleted organoids transcripts			
**Transcripts increased by > 2**	**P-Value**	**Transcripts decreased by > 2**	**P-Value**
inflammatory response	3,80E-06	multicellular organism development	2,40E-08
response to lipopolysaccharide	3,20E-05	axon guidance	8,20E-07
extracellular matrix organization	7,80E-05	embryonic skeletal system morphogenesis	2,40E-06
cholesterol homeostasis	8,10E-05	anterior/posterior pattern specification	5,40E-06
superoxide metabolic process	2,90E-04	angiogenesis	6,60E-05
response to nutrient	3,50E-04	negative regulation of cell proliferation	9,80E-05
oxidation-reduction process	3,50E-04	odontogenesis	2,50E-04
antigen processing and presentation	4,00E-04	embryonic skeletal system development	3,40E-04
immune system process	4,20E-04	nervous system development	5,80E-04
sodium ion transport	6,70E-04	brain development	8,60E-04
very long-chain fatty acid metabolic process	1,00E-03	facial nerve structural organization	1,10E-03
lipoprotein metabolic process	1,10E-03	positive regulation of oligodendrocyte differentiation	1,60E-03
ion transport	1,10E-03	cellular amino acid biosynthetic process	1,90E-03
leukocyte cell-cell adhesion	1,10E-03	negative regulation of hydrogen peroxide-induced cell death	1,90E-03
retinol metabolic process	1,10E-03	negative regulation of cell migration	2,10E-03
regulation of systemic arterial blood pressure by renin-angiotensin	1,30E-03	thyroid gland development	2,20E-03
transmembrane transport	1,30E-03	lipid metabolic process	2,40E-03
lipid transport	1,80E-03	rhombomere 3 development	2,70E-03
long-chain fatty acid metabolic process	1,80E-03	vascular endothelial growth factor signaling pathway	2,70E-03

B-

GOTERM_BP_DIRECT of *Hdac2* deleted organoids transcripts			
**Transcripts increased by >2**	**P-Value**	**Transcripts decreased by >2**	**P-Value**
response to virus	1,90E-05	multicellular organism development	1,60E-08
negative regulation of viral genome replication	6,00E-05	embryonic skeletal system morphogenesis	1,90E-04
inflammatory response	7,10E-05	coronary vasculature development	2,30E-04
defense response to virus	1,70E-04	anterior/posterior pattern specification	3,20E-04
angiogenesis	1,80E-04	axon guidance	4,80E-04
positive regulation of smooth muscle cell proliferation	2,10E-04	lung-associated mesenchyme development	5,30E-04
lipid transport	4,10E-04	response to interferon-gamma	6,10E-04
immune system process	5,00E-04	vasculogenesis involved in coronary vascular morphogenesis	1,10E-03
leukocyte cell-cell adhesion	5,20E-04	angiogenesis	1,20E-03
retinol metabolic process	5,20E-04	positive regulation of gene expression	1,30E-03
response to lipopolysaccharide	5,20E-04	vascular endothelial growth factor signaling pathway	1,40E-03
transmembrane transport	9,10E-04	definitive hemopoiesis	2,40E-03
positive regulation of macrophage derived foam cell differentiation	1,00E-03	regulation of transcription, DNA-templated	3,10E-03
actin polymerization or depolymerization	1,00E-03	kidney development	3,80E-03
extracellular matrix organization	1,70E-03	embryonic skeletal system development	4,50E-03
oxidation-reduction process	1,70E-03	branching involved in ureteric bud morphogenesis	6,10E-03
negative regulation of peptidase activity	2,10E-03	telencephalon regionalization	6,10E-03
positive regulation of fever generation	2,70E-03	regulation of macrophage activation	6,10E-03
lipid catabolic process	3,30E-03	morphogenesis of an epithelium	6,30E-03

Supplementary Figure S6

A-

GOTERM_BP_DIRECT of HDAC1 deleted organoids proteins			
**Proteins increased by >0,5**	**P-Value**	**Proteins decreased by >0,5**	**P-Value**
oxidation-reduction process	2,40E-15	liver development	5,80E-05
metabolic process	8,30E-15	glutamine metabolic process	3,60E-04
lipid metabolic process	1,70E-09	metabolic process	4,60E-04
fatty acid metabolic process	2,90E-07	ATP-dependent chromatin remodeling	7,10E-04
very long-chain fatty acid metabolic process	5,20E-06	RNA splicing	1,70E-03
retinoid metabolic process	1,10E-05	response to amine	1,70E-03
lipid homeostasis	1,30E-05	protein homotetramerization	1,90E-03
fatty acid beta-oxidation	1,90E-05	response to amino acid	2,10E-03
carbohydrate metabolic process	2,60E-05	mRNA processing	2,10E-03
cholesterol metabolic process	5,60E-05	cell-cell adhesion	2,30E-03
NADH metabolic process	8,70E-05	urea cycle	2,60E-03
lipoprotein metabolic process	9,30E-05	protein homooligomerization	2,80E-03
cholesterol homeostasis	1,10E-04	oxidation-reduction process	3,10E-03
biosynthetic process	1,70E-04	response to toxic substance	3,30E-03
lipid transport	1,90E-04	retinoic acid metabolic process	4,90E-03
response to ethanol	2,60E-04	glutathione metabolic process	5,00E-03
desmosome assembly	3,30E-04	response to drug	1,00E-02
fatty acid beta-oxidation using acyl-CoA dehydrogenase	3,70E-04	covalent chromatin modification	1,20E-02
steroid metabolic process	4,30E-04	cellular amino acid biosynthetic process	1,30E-02
cholesterol transport	4,30E-04	short-chain fatty acid catabolic process	1,40E-02

B-

GOTERM_BP_DIRECT of HDAC2 deleted organoids proteins			
**Proteins increased by >0,5**	**P-Value**	**Proteins decreased by >0,5**	**P-Value**
oxidation-reduction process	2,70E-09	glutathione metabolic process	8,20E-05
metabolic process	7,10E-08	protein homooligomerization	3,30E-04
glutathione metabolic process	2,00E-06	response to selenium ion	9,60E-04
response to drug	1,40E-05	cell-cell adhesion	2,00E-03
peptide catabolic process	1,10E-04	oxidation-reduction process	4,40E-03
response to nutrient	2,60E-04	metabolic process	6,10E-03
biosynthetic process	5,20E-04	cellular response to interleukin-4	7,10E-03
response to hormone	1,10E-03	phosphorylation	8,00E-03
response to activity	1,20E-03	response to cadmium ion	8,20E-03
response to ethanol	1,60E-03	response to amino acid	1,20E-02
response to oxidative stress	2,50E-03	regulation of eIF2 alpha phosphorylation by heme	2,30E-02
lipid metabolic process	3,40E-03	response to L-ascorbic acid	2,30E-02
cellular amino acid metabolic process	4,60E-03	response to oxidative stress	2,50E-02
response to cadmium ion	6,70E-03	response to methylmercury	3,20E-02
skeletal muscle satellite cell migration	8,50E-03	heart development	3,50E-02
cysteine metabolic process	8,50E-03	response to herbicide	3,70E-02
cellular response to thyroxine stimulus	8,50E-03	regulation of stress-activated MAPK cascade	3,70E-02
positive regulation of lipase activity	1,70E-02	oxygen transport	4,60E-02

Supplementary Figure S7

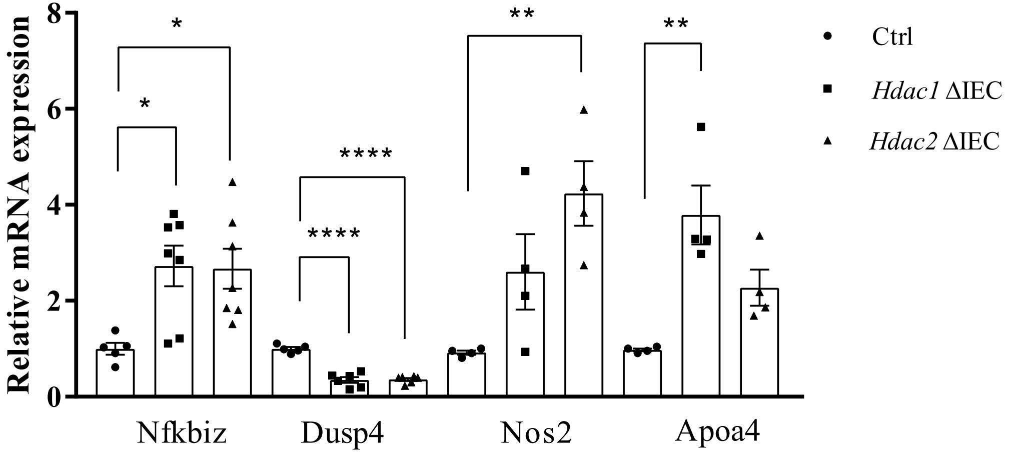

Supplementary Figure S8. Western Blot data

Figure 1 B

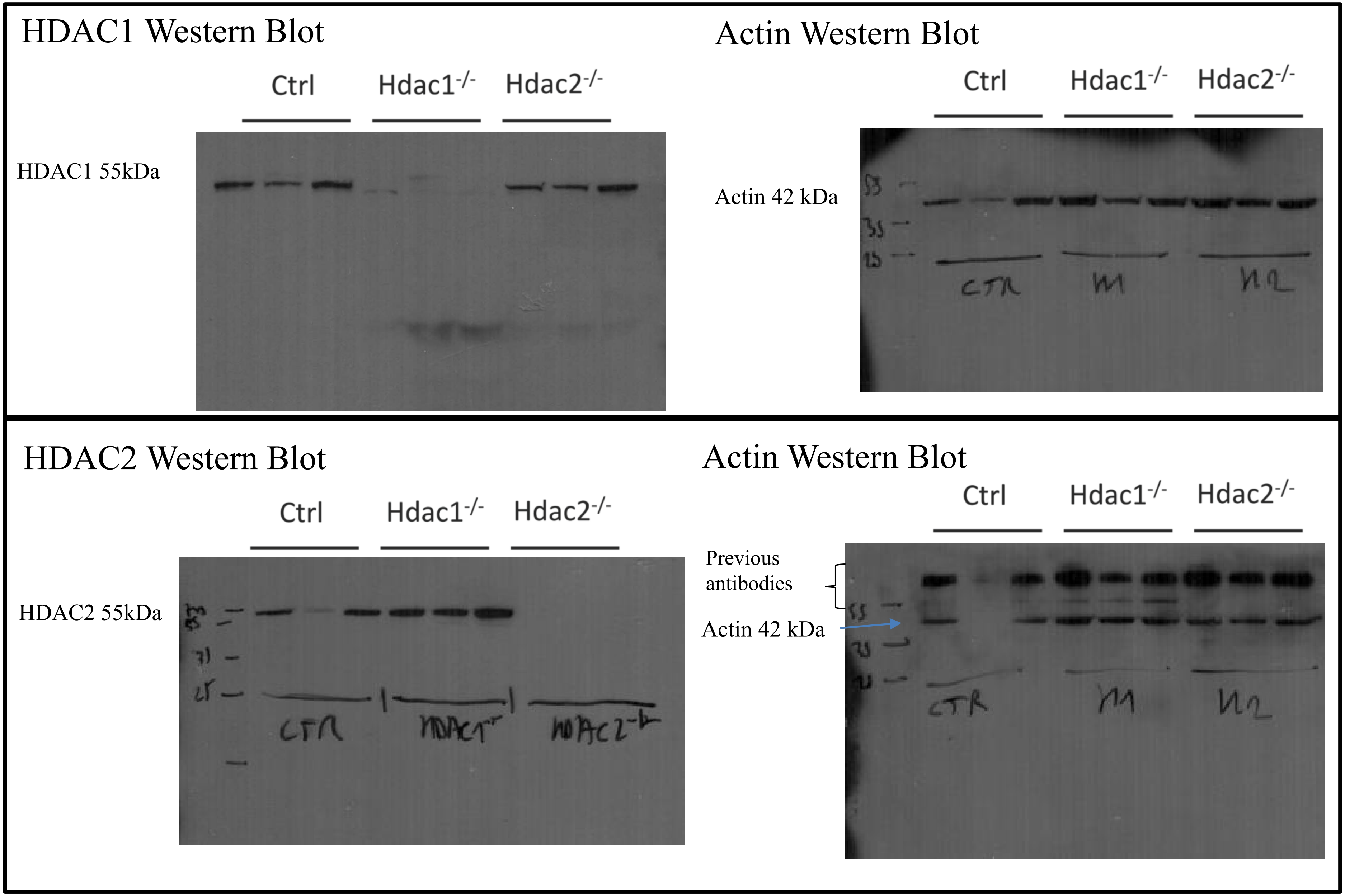

Figure 7 A

Phospho Stat3 Western Blot

Ctrl | Hdac1$^{-/-}$ | Hdac2$^{-/-}$

Stat3-P
79-86 kDa

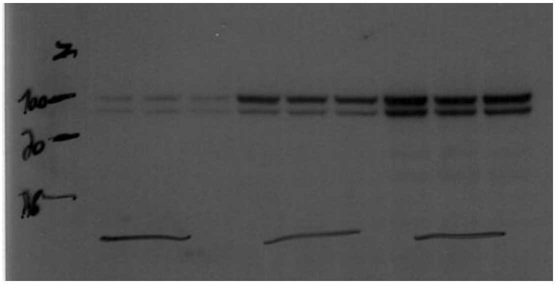

Total Stat3 Western Blot

Ctrl | Hdac1$^{-/-}$ | Hdac2$^{-/-}$

Stat3-tot
79-86 kDa

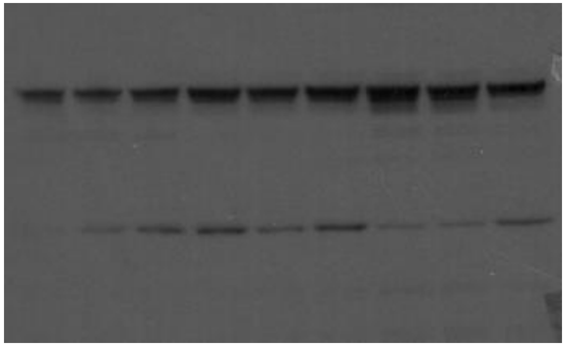

Actin WesternBlot

Ctrl | Hdac1$^{-/-}$ | Hdac2$^{-/-}$

Actin 42 kDa

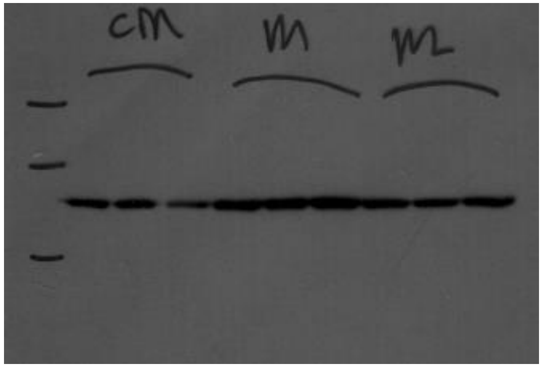

Figure 7 C

HMGCS2 Western Blot

HMGCS2 50 kDa

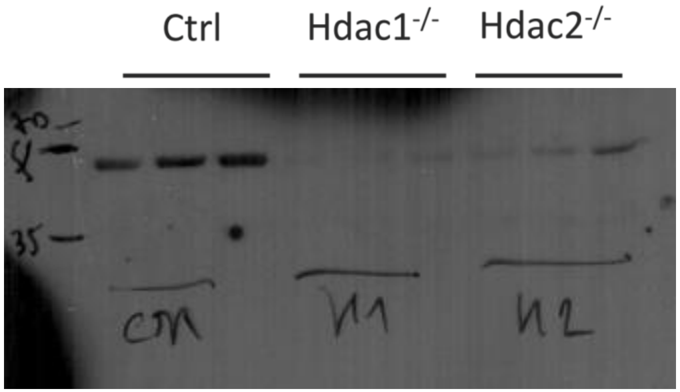

Actin Western Blot

Actin 42 kDa

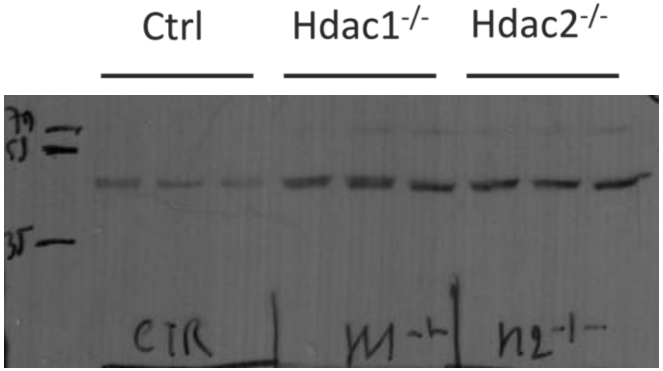

Supplement: Supplementary file 2 — Supplementary Figures S1–S8 [file 41598_2019_41842_MOESM2_ESM.pdf]
